# Prevalence of Self-Reported Musculoskeletal Disorders in Dentists—A Cross-Sectional Study in Portugal and Italy

**DOI:** 10.3390/healthcare13091020

**Published:** 2025-04-28

**Authors:** Alessandro Bracciale, Maria Conceição Manso, Francesca Bracciale, Liliana Gavinha Costa

**Affiliations:** 1 Faculty of Health Sciences, University Fernando Pessoa, 4200-150 Porto, Portugal; alessandrobracciale593@gmail.com (A.B.); francescabracciale1@gmail.com (F.B.); 2 RISE-Health, Faculty of Health Sciences, University Fernando Pessoa, Fernando Pessoa Teaching and Culture Foundation, 4200-150 Porto, Portugal; 3 LAQV/REQUIMTE, University of Porto, 4050-453 Porto, Portugal

**Keywords:** musculoskeletal disorders, endodontics posture, ergonomics, Nordic Musculoskeletal Questionnaire (NMQ), working habits

## Abstract

**Objectives**: A dentist may assume incorrect working positions throughout their professional career, which can lead to musculoskeletal disorders (MSDs). This study aimed to quantify the prevalence of self-reported MSDs in dentists and the body region most affected, as well as to evaluate the most frequent working position, the area of work and the age group most frequently associated with MSDs. **Methods**: This is a cross-sectional observational study adhering to the STROBE and CHERRIES guidelines, approved by the local Ethics Committee. An online questionnaire (including the Nordic Musculoskeletal Symptoms Questionnaire) was applied to a convenience sample of Portuguese and Italian dentists. Data analysis was conducted with non-parametric comparisons (IBM© SPSS^®^ Statistics vs. 28.0, *p* < 0.05). **Results**: A total of 341 dentists (170 Portuguese, 171 Italian) aged 18–75 years participated. The prevalence of self-reported MSDs in Portugal and Italy was 78.8% and 81.9%, respectively. The most affected self-reported MSD body regions were the neck (65.3% Portuguese and 61.4% Italian dentists), shoulder (49.4% Portuguese and 39.2% Italian) and lumbar region (52.4% and 39.2%, respectively). The neck region was the one reported to cause the highest work absenteeism. The higher age group (41–50 years and higher) has a higher risk for self-reported MSD. Multivariate analysis highlighted increased practice time as a significant risk factor for MSDs in the previous year in both countries. Age group, practice duration and work position relative to the patient were significantly linked to MSD occurrence and work avoidance. **Conclusions**: Dentists showed a high prevalence of self-reported MSD despite performing clinical activity mostly seated. It was concluded that dentists older than 40 years and those who have been working for more than 20 years have a higher risk of MSD, and that endodontics is the area where they report feeling more discomfort. Dentists should be encouraged to take more breaks between appointments and regular exercise to prevent the development/progression of MSDs.

## 1. Introduction

Dentistry is a demanding field of medicine requiring significant physical and mental work from its practitioners. Dentists frequently perform intricate therapeutic procedures within confined spaces, demanding high levels of concentration and precision. These tasks necessitate maintaining muscle equilibrium and adopting asymmetrical postures for extended periods, often resulting in significant physical strain [1,2]. In particular, the unnatural positions adopted to optimize visualization during procedures (in this context, they have been identified as including neck tilt/rotation, forward bending resulting in loss of cervical and lumbar lordosis, and elevated arms during prolonged static isometric/excentric contraction) can lead to musculoskeletal disorders (MSDs), with prevalences ranging from 88.9% for general dentists and 83.6% for orthodontists in countries such as Australia [3] to 84.6% for Italian dentists [4].

The Nordic Musculoskeletal Symptoms Questionnaire (NMSQ) is widely used in epidemiological studies to assess the prevalence of MSDs across various occupational groups. Developed in 1987 as part of a Nordic Council of Ministers initiative, the NMSQ provides a standardized tool for evaluating MSD prevalence and screening in occupational health contexts [5]. Over time, it has become a valuable resource for studying MSDs in dental professionals [4,6,7,8,9,10,11]. Ergonomics has emerged as a critical discipline in addressing the challenges of MSDs in dentistry. By optimizing the interaction between dentists, their tools, and the operative environment, ergonomics enhances work quality, reduces task duration, and promotes the health and well-being of practitioners [12]. The consequences of MSDs, including reduced productivity, decreased job satisfaction, and even the risk of early retirement, underscore the importance of ergonomic interventions [9,12,13]. Despite the availability of ergonomic solutions, the prevalence of MSDs among dentists remains alarmingly high [10,11,14], with a recent meta-analysis reporting rates ranging from 19.4% to 100% [15] and 15.9% experiencing limitations in daily activities [9].

Dentists in the target countries of our study have already been identified as being at ergonomic risk, and their habits may influence the development of MSDs [16,17]. In Portugal, dental professionals have high levels of stress and MSD complaints in the neck, back, shoulders and wrists, with a correlation between stress and the prevalence of MSD complaints in the last 7 days (r = 0.30; *p* < 0.01) and in the last 12 months (r = 0.28; *p* < 0.01), with female professionals reporting higher levels of stress (mean = 18.14) than their male colleagues (mean = 16.89), with a statistically significant difference (*p* = 0.03) [17]. Musculoskeletal symptoms are present in a high proportion of the population, with associated pathologies diagnosed in 5.4% [18].

Although Gandolfi and colleagues studied MSDs in Italian dentists and dental hygienists [4], there are few studies reported for the Portuguese reality [17,18]. Building on previous studies that utilized the Nordic questionnaire [4,6,7,8,10,11,19,20]—particularly those modified to include questions about diagnosed pathologies [10] and methods for assessing posture [11,19]—the present study incorporates additional factors. These include the presence or absence of breaks between appointments, the use of visual aids, the dominant working hand, the preferred working position, and the most common working posture in relation to the patient. Furthermore, this study expands upon earlier research by identifying specific areas of dental activity associated with the prevalence of musculoskeletal disorders (MSDs), moving beyond the mere documentation of localized pain [19,20]. Unlike some prior studies that included dental assistants and students [12,19], the current research focuses exclusively on practicing dentists. Samat and associates [19] found a 44.9% (95% CI: 39.65–50.07) back pain prevalence in the past 12 months, but only 28% for dentists, and after adjusting for confounders, significant risk factors for back pain included poor posture (OR = 3.52; 95% CI: 2.22–5.59). Macrì and associates [20] accounted for an MSD prevalence of 87.2% among Italian dentists and 91.4% among Peruvian dentists. The present study, like Macrì and associates [20], is held simultaneously in two countries.

The objective of this study was to quantify the prevalence of self-reported MSDs in dentists and the body region most affected. The study also sought to evaluate the most frequent working position, the area of work and the age group most frequently associated with self-reported MSDs, as well as risk factors associated with self-reported MSDs in the previous 12 months.

To achieve these objectives, the following alternative hypotheses were proposed:

First alternative hypothesis: A significant proportion of dentists report musculoskeletal disorders (MSDs), with specific body regions (e.g., neck, shoulders, or lumbar region) being more frequently affected.
Second alternative hypothesis: Dentists who adopt specific working positions (e.g., seated, standing, or alternating) are significantly more likely to report MSDs.
Third alternative hypothesis: Certain areas of dental practice (e.g., oral surgery, endodontics) are significantly associated with higher prevalence of MSDs.
Fourth alternative hypothesis: The prevalence of MSDs differs significantly across age groups, with older dentists reporting higher prevalence.
Fifth alternative hypothesis: Specific factors such as increased practice time, prolonged work hours, lack of breaks, and lack of exercise are significantly associated with the prevalence of MSDs.

## 2. Materials and Methods

### 2.1. Type of Study and Sample

A cross-sectional observational study was conducted and followed the STROBE guidelines [21] as well as adhering to the CHERRIES guidelines [22], using a convenience sample of dentists practicing in Portugal and Italy. The sample size was calculated based on the power analysis estimation method. Given the literature’s indication of varying prevalences (self-reported or observed), it was decided to consider the worst-case scenario, in which the proportion of dentists with and without MSDs is similar (50%), irrespective of the body region or country where the measurement is intended to be conducted. The additional assumptions made were an accuracy of ±7.5% and a type I error probability of 0.05. In this case, with the expression $n=0.25\times {\left(2\times 1.96/\;CI\;width\;\right)}^{2},$ it was estimated that approximately 170 dentists should be questioned in each country. The inclusion criteria for the participants were being a dentist, aged up to 75 years old and still performing their activity. The exclusion criteria included retired professionals, students and professionals who had suffered accidents or trauma or other situations that caused permanent MSDs.

### 2.2. Data Collection Instrument

The questionnaire comprised 50 questions and was designed to take between 8 and 10 min to complete. It was divided into four sections: the first section pertained to socio-demographic issues (two questions); the second section focused on professional activity (eight questions); the third section addressed health and well-being (four questions); and the fourth section incorporated the translated and validated Nordic Musculoskeletal Symptoms Questionnaire (NMQ) [23,24] and a numeric pain scale (36 questions). The NMQ adapted the questions regarding the “last 12 months” for the context of dentistry. The questionnaire was administered in both Portuguese and Italian, to ensure its relevance to a cross-border audience. A preliminary evaluation was conducted in a local context to ascertain the questionnaire’s practicality and technical functionality. The first page with information regarding the study and the informed consent also contained the two first questions, and all questions had mandatory answers. Therefore, it is not possible to calculate the attrition rate. The completion rate was 100%.

The dissemination of the open questionnaire link was conducted through the researcher’s social networks and via Dental Medicine Forums catering to professionals from both countries, not repeating the invitation. The distribution of the questionnaire was not associated with emails that would allow the participants to be identified. The participants voluntarily agreed to complete the questionnaire. Data collection took place during April–June of 2022.

### 2.3. Ethics

The present study was approved by the Ethics Committee of Fernando Pessoa University (Porto, Portugal) with the approval reference of CE-FCS-276/22, 22 March 2022. Prior to the commencement of the questionnaire, information regarding the study objectives, length and storage of the collected data was given and the participant was requested to provide their consent (Informed Consent form) in accordance with the CHERRIES guidelines. It was stated on the Google Form website page that the survey was voluntary, and that no incentives were provided for completion, nor was any personal data collected. Authorization was obtained for the use of the Nordic Musculoskeletal Questionnaire.

### 2.4. Data Treatment

The data analysis was conducted using IBM^®^ SPSS© Statistics vs. 28.0 (IBM Corp. released 2021, Armonk, NY, USA). The descriptive analysis of qualitative variables was performed through counts and respective relative frequencies (%), while for quantitative variables, the mean and standard deviation (for comparison with other published studies) and median and respective interquartile range (Me (Q1–Q3)) due to non-normal behavior were used, as well as the range (minimum and maximum) observed. All participants’ data were complete (no missing data).

A significance value of 0.05 (95% confidence level) was considered significant to detect differences or associations between variables. Group comparisons (e.g., by country) employed the chi-square test (qualitative variables) and the Mann–Whitney U test (quantitative variables). Three variables were created to determine the prevalence of self-reported activity-related MSDs (each one accounting for the existence of at least one body zone with activity-related MSDs) in the last 12 months, the last 7 days, and activity-related MSDs in dentistry over 12 months, with 95% confidence intervals calculated using the exact method.

To identify risk or protection factors for activity-related MSDs (last 12 months and dentistry-specific), namely the preventable and the non-alterable, bivariate logistic regression was used, followed by multivariable logistic regression, using the Wald method (*p* < 0.05 to enter, *p* > 0.20 to exclude), to further assess these factors. Variables regarding feeling pain or discomfort during or after clinical activity were not included due to obvious collinearity. The quality of the multivariate analysis was evaluated by calculating the area under the ROC curve (AUC).

## 3. Results

In total, 341 dentists from Portugal (170) and Italy (171) voluntarily participated in the study (all of which fully completed the questionnaire), of both sexes, aged 23–72 years old.

The study (Table 1) highlights key demographic and professional differences between Portuguese and Italian dentists. A significantly higher percentage of Portuguese respondents were female (61.2% vs. 48.5%, *p* = 0.019). Portuguese dentists were younger (mean age 37 vs. 41.2 years, *p* = 0.022) and had practiced dentistry for fewer years (mean 11.5 vs. 14.8 years, *p* = 0.048).

Notable differences in areas of practice were observed among study participants (*p* = 0.001). Portuguese dentists predominantly specialized in oral surgery (21.2%) and endodontics (21.2%), while Italian dentists focused on orthodontics (21.6%), endodontics (15.6%), and oral surgery (14.6%). Periodontology was significantly more common among Italian dentists (12.3% vs. 1.2%, *p* = 0.001).

Italian dentists were more likely to work 25 h or less per week, while Portuguese dentists worked 45 or more hours more frequently (*p* < 0.001). Weekly physical activity distribution was similar between groups (*p* = 0.321).

### 3.1. Prevalence of Self-Reported MSDs in Dentists and the Body Region Most Affected

A high prevalence of self-reported MSDs in any part of the body was reported in both groups over the past year: 78.8% of Portuguese dentists and 81.9% of Italian dentists (Table 2, including 95% confidence interval self-reported prevalence estimation). The neck, shoulder and lumbar regions were most affected, with similar patterns across both groups. Notably, Portuguese dentists reported higher rates of work-disrupting pain in the elbow (26.3% vs. 17.9%) and wrist/hand regions (32.2% vs. 18.4%).

In the past seven days, 58.2% of Portuguese and 56.7% of Italian dentists reported MSDs (Table 2 and Table 3).

The neck was the most commonly affected region in both countries (65.3% of Portuguese and 61.4% of Italian dentists, *p* = 0.456), but Portuguese dentists reported higher pain levels (median 6 vs. 5, *p* = 0.001).

Significant differences were observed in the shoulder region: Italians reported more cases involving both shoulders (25.7%), while Portuguese dentists reported higher prevalence in the right shoulder (23.5%). Lumbar region prevalence was similar (52.4% vs. 50.9%), but Portuguese dentists reported significantly higher pain levels (*p* = 0.034).

Portuguese dentists experienced more wrist and hand pain, though no significant differences in prevalence were noted (64.7% vs. 78.4%, *p* = 0.046). In the ankle and foot region, prevalence was low in both groups (11.8% vs. 12.3%, *p* = 0.599), but Italian dentists reported higher pain intensity (median 5 vs. 4, *p* = 0.030).

Work avoidance due to pain was most associated with the neck in both countries (15.9% of Portuguese vs. 9.4% of Italians). Differences were observed in the shoulder (right shoulder more reported by Portuguese) and wrist/hand regions (left more commonly reported in Portugal).

### 3.2. Working Position, the Area of Work and the Age Group Most Frequently Associated with Self-Reported MSDs

The seated working position was the predominant posture (Table 4) in both countries (47.1% of Portuguese vs. 54.4% of Italians, *p* = 0.347). Italian dentists were significantly more likely to take breaks between consultations (57.3% vs. 40%, *p* = 0.001). Portuguese dentists predominantly used their right hand (92.4%), whereas Italians showed a higher prevalence of left-hand dominance (7.6% vs. 1.8%, *p* = 0.001). Vision aid usage varied significantly between countries (*p* < 0.001). Among Portuguese dentists, 74.7% did not use vision aids, while 25.3% employed microscopes. In contrast, 47.4% of Italian dentists used magnifying glasses, 5.3% microscopes, and 47.4% did not use vision aids. Portuguese dentists reported significantly more pain or discomfort during or after clinical work (*p* = 0.045). Oral surgery was the most discomfort-inducing activity in Portugal, while endodontics was the primary source in Italy (no significant difference). Periodontology was reported significantly more often in Italy (16.4% vs. 2.2%, *p* = 0.035).

### 3.3. Risk Factors Associated with the Presence of Self-Reported MSD in the Last 12 Months and the Presence of Self-Reported MSD While Avoiding the Routine Work for the Same Period

Regarding risk and protective factors for MSDs (Table 5), in Portugal, female dentists were identified as the group more prone to be at significant risk for MSDs in the past 12 months (OR = 2.83, 95% CI: 1.06–7.57). Increased practice time was associated with higher MSD prevalence (*p* = 0.027), while regular exercise for 5–10 years appeared to be a protective factor (OR = 0.32, 95% CI: 0.096–1.07), though not significant. Longer practice times (11–19 years and ≥20 years) further increased risk, albeit non-significantly.

Dentists working 36–44 h weekly were 11 times more likely to report MSDs than those working ≤25 h (OR = 10.70, 95% CI: 2.06–55.49, *p* = 0.013). Not having breaks between appointments (OR = 3.02, 95% CI: 1.14–7.99) and working while seated (OR = 7.06, 95% CI: 2.01–24.86) or alternating between seated and standing positions (OR = 7.86, 95% CI: 2.10–29.47) are significant risk factors.

In Italy, increased practice time was the only significant risk factor for MSDs (*p* = 0.037). Practice time of 11–19 years or ≥20 years increased the risk (OR = 1.75 and OR = 2.63, respectively), consistent with findings from Portugal.

Regarding self-reported MSD-related work avoidance (Table 6), data show that in Portugal, dentists aged 41–50 years had a higher likelihood of work avoidance due to MSDs (OR = 3.70, 95% CI: 1.07–12.81). Dentists practicing for 11–19 years were six times more likely to avoid work compared to those with ≤4 years of experience (OR = 5.99, 95% CI: 1.51–23.73).

Exercise frequency was also linked to MSD risk. Dentists exercising ≥3 times weekly had a significantly higher risk (OR = 4.15, 95% CI: 1.37–12.62) compared to those who did not exercise, while exercising 1–2 times weekly showed a non-significant increase in risk (OR = 2.89, 95% CI: 0.86–9.69, *p* = 0.020).

Age group, practice duration and work position relative to the patient were significantly linked to MSD occurrence and work avoidance. Dentists aged 31–40 years exhibited a markedly higher risk of MSDs compared to younger counterparts (OR = 38.28, 95% CI: 1.49–982.75).

Regarding age, practice duration and work position (Table 6), Italian dentists aged 41–50 years faced a tenfold higher risk of MSDs compared to younger dentists (OR = 10.27, 95% CI: 0.91–115.83), while those aged ≥51 years had a threefold increased risk (OR = 3.29, 95% CI: 0.84–12.82). Dentists with ≥20 years of practice exhibited the highest risk, approximately 54 times greater than those with ≤4 years of experience (OR = 54.02, 95% CI: 2.11–1382.75).

Dentists working behind the patient experienced an 80% reduction in MSD risk compared to those working to the patient’s right (OR = 0.20, 95% CI: 0.04–0.95).

Principal dentistry clinical activity was not associated with the presence of self-reported MSD in the last 12 months (Table 5) and the presence of self-reported MSD while avoiding the routine work for the same period (Table 6) for both groups. Academic training in ergonomics for clinical practice was a significant risk factor for self-reported MSD in the last 12 months among Portuguese dentists in the bivariate analysis (Table 5); however, this association was not significant in the multivariate model.

## 4. Discussion

### 4.1. Discussion of Current Findings

The present study set out to quantify the prevalence of self-reported musculoskeletal disorders (MSDs) among dentists in Portugal and Italy and to identify the most affected body regions. Additionally, the study aimed to evaluate the impact of working position, area of work, age group, and other risk factors associated with MSDs in the past 12 months.

The findings confirm the first alternative hypothesis, as a significant proportion of dentists in both countries reported MSDs. Specifically, 78.8% of Portuguese dentists and 81.9% of Italian dentists experienced MSDs over the past year, with the neck, shoulder, and lumbar regions being the most affected body parts. Moreover, fists/hands are the main body area reported for avoiding the dentistry work routine due to MSD in the last 12 months, at 32.2% and 18.4%. Pain severity differed between countries, with Portuguese dentists reporting significantly higher levels in the neck and lumbar regions and significantly less in the ankle and foot compared to their Italian counterparts.

The global probability of female dentists developing MSDs was 2.83 times higher than that of their male counterparts, and although this finding aligns with numerous studies that have demonstrated a higher prevalence of work-related MSDs among women [15,25,26], one may also argue this can be linked with a possibility of female dentists being more likely to respond to surveys (the sample comprised 61.2% Portuguese female dentists, but only 48.5% Italian female dentists), rather than being more prone to musculoskeletal disorders. As the present study is based on self-report, the present doubt remains unresolved. Nevertheless, this disparity can be attributed, at least in part, to the differing physical constitutions between the sexes. Furthermore, hormonal changes, a higher incidence of osteoporosis and additional physical stress, also attributed to the development of physiological and physical illnesses specific to the female gender, are also a contributing factor. However, the literature on the anatomical sites affected remains inconclusive [26].

It was assessed that 78.8% of Portuguese and 81.9% of Italian dentists self-reported MSD in the previous 12 months. This finding is consistent with the results of previous studies that have reported comparable prevalence rates, such as Leggat and Smith [27], reporting a prevalence of MSD of 87.2% in both genders in a study of 285 Australian dentists. A similar prevalence has been documented in China, with a study by Yi and associates [28] reporting an 85.6% prevalence of MSD. In a sample of dentists of Islamabad, Pakistan, the overall prevalence of work-related musculoskeletal disorders was significantly higher, at 91.5% (95% CI: 87.5–95.9%), but it included individuals with a history of injury to the neck or shoulder [29], which might explain the elevated prevalence. Despite the growing awareness and training in ergonomics, the prevalence of MSDs remains high, indicating a lack of respect for this important issue.

In response to the query regarding the impact of MSDs on professional activities over the past 12 months, 28.8% of Portuguese and 19.3% of Italian dentists of both genders reported experiencing one or more MSDs, which had a temporary impact on their routine, hindering their professional output [30]. It is further reported that one-third of dentists were compelled to alter their professional trajectory, whilst a further two-thirds have experienced MSD at least once in their lifetime.

Amongst the dentists who completed this questionnaire, 58.2% of Portuguese and 56.7% of Italian dentists reported the presence of MSD in the previous seven days. A recent study conducted in Germany showed that about 60% of dentists experienced one or more MSDs in the last 7 days [31], which allows us to identify clear similarities between professionals from different countries.

With regard to the most self-reported anatomical regions of the dentists in the countries under observation, the following were identified: neck (59.9%), shoulders (43.3%) and lower back (37.7%). Gandolfi and associates [4] also identified that the most affected regions were the neck (59.9%), followed by the lumbar region (52.1%), the shoulder (43.3%) and the back region (37.7%), showing also a high prevalence of pain. The present study’s findings on the high prevalence of MSDs in the cervical region are consistent with those of a study conducted in Brazil, which involved 204 dentists who reported that 81.4% of the study participants experienced neck pain [32].

The second alternative hypothesis, that specific working positions influence the prevalence of MSDs, was partially supported. While the seated position was the most prevalent among dentists in both countries, those working in seated or alternating seated/standing positions were at significantly greater risk of developing MSDs. Notably, lack of breaks between appointments emerged as a key factor, with Portuguese dentists less likely to take breaks compared to Italians (40% vs. 57.3%, *p* = 0.001). Concerning the working position parameter, Portuguese dentists working in a seated position and alternating between standing and seated positions were, respectively, 7.06 times more likely (seated) and 7.86 times more likely (alternating standing and sitting) to develop musculoskeletal disorders (MSDs) than those standing. Operators tend to work in incorrect ergonomic positions, even when seated. They assume singular and asymmetrical positions, flexing their heads forward and swiveling to the side, with arms outstretched [32]. These postures, if maintained for extended periods over several days, can result in muscle and joint overload. The field of ergonomics aims to mitigate cognitive and physical stress, prevent occupational diseases associated with dentistry, and enhance productivity, thereby improving both the quality of care and the patient’s experience [33]. Most dental professionals surveyed engaged in physical activity (70.1%), but only a few had satisfactory knowledge of ergonomic guidelines (12.7%) [4]. The most common factor identified as a contributing factor to the development of MSDs appears to be prolonged static posture, which has been shown to induce a high static muscle load on the neck and shoulder areas [3]. The prevalence of WMSDs was correlated with the number of hours worked per day and week, with a higher risk among those working more than 5 h per day and more than 30 h per week [4]. The risk of developing MSD is further increased due to long working hours and when performing precision movements and eye–hand coordination [34]. This finding lends support to the hypothesis that extended working hours, as evidenced in our study (>40 h per week), may act as an exacerbating risk factor. Operators who work for extended periods, particularly in the absence of interruptions, have been observed to be more susceptible to the development of MSD [35].

The use of aids, such as magnifying glasses and microscopes, in clinical practice has been the subject of evaluation. It was observed that Portuguese dentists reported the use of these aids less frequently than their Italian counterparts. As demonstrated by [36], the use of visual aids, notably the microscope, led to a substantial decrease in neck flexion and a significant positive impact on the reduction in MSD.

The third alternative hypothesis, positing that certain areas of dental practice are associated with higher MSD prevalence, was also confirmed. Dentists specializing in oral surgery and endodontics reported higher rates of discomfort, with notable differences in the most discomfort-inducing activities: oral surgery for Portuguese dentists and endodontics for Italians. Periodontology was more commonly reported in Italy, potentially reflecting differing practice patterns or ergonomic adaptations in the two countries. A significant proportion of the dentists participating in the study were endodontists (21.2% Portuguese and 15.8% Italian) and oral surgeons (21.2% Portuguese and 14.6% Italian). Studies indicate that endodontics has a high propensity to develop MSD, as reported by Zarra and Lambrianidis [37] with the fact that 61% of the Greek endodontists had suffered from musculoskeletal disorders (MSDs) in the last 12 months, and of those, 69% reported pain in more than one part of the body. Aghahi and associates [38] state that for dental students, the clinical areas with the most pain were endodontics and periodontics.

The fourth alternative hypothesis, suggesting that MSD prevalence varies across age groups, was strongly supported. The present study observed that dental practitioners who have been working for more than 20 years are 3.85 times more likely to suspend activity due to MSDs. Age and practice duration were significant risk factors in both countries. Portuguese dentists aged 41–50 years and Italian dentists aged ≥51 years reported higher MSD prevalence, aligning with findings that prolonged practice time increases the risk of MSDs. These findings are somewhat divergent with other research that has shown an association between age and the prevalence of MSDs, with a higher prevalence found among professionals with 2–5 years of experience after graduation [4], and the highest prevalence observed among those with 5–10 years of professional experience [29,39], and as the report of an early development of MSDs in the dental profession [28].

Finally, the fifth alternative hypothesis, which linked specific risk factors such as extended practice time, prolonged work hours, and lack of exercise to MSD prevalence, was partially confirmed. Extended practice time significantly increased the likelihood of MSDs in both countries. However, the relationship between exercise and MSDs showed unexpected results, with dentists exercising ≥3 times weekly reporting a higher risk of MSDs. This finding may indicate compensatory physical activity among those already experiencing MSDs rather than a true protective effect.

The present study observed that 52.4% of Portuguese and 47.4% of Italian dentists did not engage in physical activity. Contrary to our initial hypothesis, our findings indicated that physical activity was associated with an increased risk of developing MSDs. This phenomenon can be explained by the attempt of dentists who already have MSDs to stop their progression. The study of Benfaida and associates [40] observed a correlation between MSD and factors such as lack of physical activity (40.3% of participants did not practice sport), as well as other lifestyle factors such as being overweight, in addition to years of practice. The cross-sectional nature of the present study precludes definitive conclusions regarding the temporal sequence of MSD occurrence and the potential role of physical exercise in mitigating its progression. This fact could be considered in future studies to ascertain whether the increased demand for physical exercise is attributable to a necessity to mitigate the progression of injuries.

### 4.2. Study Limitations

This study provides valuable insights into the self-reported prevalence and associated factors of musculoskeletal disorders (MSDs) among Portuguese and Italian dentistry professionals but has several limitations. Its cross-sectional design prevents establishing causal relationships between risk factors (e.g., ergonomic practices, physical activity, or years of experience) and MSD prevalence, leaving the temporal sequence of MSD development hypothetical. The reliance on self-reported data introduces potential recall bias and subjective interpretation, possibly leading to over—or underestimation of MSD prevalence. Differences in participants’ understanding or reporting of symptoms further complicate accuracy.

While the sample size was considerable, it may not fully represent all dentistry professionals in Portugal and Italy due to gender imbalances (e.g., a higher percentage of female participants) and professional specialization clusters (e.g., endodontists and oral surgeons). This study aimed to recruit a representative sample of dentists from both Italy and Portugal, but as with many survey-based studies, participant recruitment was subject to response rates and practical limitations. However, statistical power is not solely determined by sample size but also by the strength of associations observed, and several medium and large effect sizes (OR values above 2 and 3, respectively) [41] maintained methodological rigor in data collection and interpretation.

The absence of objective ergonomic assessments or physical examinations to validate self-reported MSDs limits the study, as direct observational or biomechanical analyses, though resource-intensive, could have strengthened the findings.

The relationship between physical activity and MSDs must be interpreted cautiously, as the study did not differentiate exercise types, intensities, or frequencies, potentially reflecting confounding factors such as compensatory physical activity by those already experiencing MSDs. Psychosocial stressors, including work-related stress, job satisfaction, and mental health—key contributors to MSDs—were not assessed.

Cultural, systemic, and healthcare differences between Portugal and Italy, such as variations in ergonomic training, healthcare access, or preventive measures, were not explored and may affect the comparability of results. Lastly, while common anatomical regions affected by MSDs were identified, the study did not investigate the severity, chronicity, or impact of comorbidities, limiting the scope of its conclusions.

### 4.3. Future Research Directions

Future research should aim to address these limitations by employing observational longitudinal designs, incorporating objective ergonomic and biomechanical assessments, using among other tensiomyography [42,43], and exploring the influence of psychosocial and cultural factors. Additionally, expanding the sample to include a more diverse and representative cohort of dental professionals could further enhance the generalizability of the findings.

### 4.4. Clinical Implications

This study holds important practical, theoretical, and scientific relevance.

Practically, it reveals a high prevalence of self-reported MSDs (78.8% in Portugal, 81.9% in Italy), with the neck, shoulders, and lower back most affected. The neck was linked to the most absenteeism, emphasizing the need for targeted ergonomic strategies. Risk factors such as long practice time, poor posture, and infrequent breaks—especially in Portugal—highlight areas for intervention. The impact on dentists’ quality of life underscores the urgency of education on ergonomic practices and preventive measures.

Theoretically, the study confirms the link between MSDs and working posture, while challenging assumptions about the benefits of frequent exercise. Cross-country comparisons reveal differences in work routines and specialization, enriching existing models. It also supports the NMSQ as a valid tool in dental research.

Scientifically, it contributes new data on occupational health in dentistry and identifies directions for future research, including long-term and psychosocial studies. The findings can inform more effective ergonomic interventions in both countries.

## 5. Conclusions

This study highlights the significant burden of musculoskeletal disorders (MSDs) among dentists in Portugal and Italy, with over 78% of professionals in both countries self-reporting MSDs within the past year. The neck, shoulder, and lumbar regions were the most commonly affected areas, with Portuguese dentists experiencing higher pain severity in the neck and lumbar regions compared to their Italian counterparts. These findings underscore the physical toll of dentistry, exacerbated by prolonged working hours, lack of breaks, and ergonomically challenging positions.

The study also revealed notable differences between the two countries. Portuguese dentists were more likely to work extended hours and less likely to take breaks, factors that may contribute to their higher reported pain severity. Conversely, Italian dentists more frequently used vision aids, such as magnifying glasses, which could mitigate certain ergonomic risks.

Key risk factors identified include prolonged practice time, specific working positions, and lack of ergonomic interventions. Unexpectedly, dentists exercising frequently were at higher risk of MSDs, potentially indicating compensatory activity among those already affected. Age and practice duration were also significant predictors, with older and more experienced dentists reporting higher prevalence and severity of MSDs.

These findings emphasize the urgent need for targeted ergonomic interventions, regular breaks, and other preventive strategies tailored to dentists’ specific needs. While this study provides valuable insights, its limitations, such as reliance on self-reported data and lack of objective ergonomic assessments, should be considered.

It lays the groundwork for future research and intervention. It is recommended that dentists be encouraged to take breaks between appointments and engage in regular exercise to prevent various musculoskeletal problems. The promotion of healthy attitudes, attentiveness to bodily signals, and consideration for the needs of the body are proposed as novel pedagogical approaches to enhance the well-being of both body and mind.

## Figures and Tables

**Table 1:** Comparison of social demographic and work variables of the Portuguese and Italian dentists.

Variables	Characteristics/ Statistics	Portuguese Dentists	Italian Dentists	*p*
Gender	Female	104 ^a^ (61.2%)	83 ^b^ (48.5%)	0.019 *
	Male	66 ^b^ (38.8%)	88 ^a^ (51.5%)	
Age (years)	Median (Q1–Q3)	35 ^b^ (27.75–42)	37 ^a^ (28–56)	0.022 **
	Min–Max	23–72	24–70	
	Average (St.Dev)	37 (11.3)	41.2 (14.3)	
	≤30 years	63 (37.1%)	64 (37.4%)	
	31–40 years	56 (32.9%)	31 (18.1%)	
	41–50 years	28 (16.5%)	24 (14%)	
	≥51 years	23 (13.5%)	52 (30.4%)	
Practice time as dentist (years)	Median (Q1–Q3)	9.5 ^b^ (2–17)	10 ^a^ (3–28)	0.048 **
	Min–Max	1–49	1–44	
	Average (St.Dev)	11.5 (10.3)	14.8 (12.9)	
	≤4 years	59 (34.7%)	61 (35.7%)	
	5–10 years	35 (20.6%)	25 (14.6%)	
	11–19 years	44 (25.9%)	22 (12.9%)	
	≥20 years	32 (18.8%)	63 (36.8%)	
Principal dentistry clinical activity	Oral surgery	36 (21.2%)	25 (14.6%)	0.001 *
	Endodontics	36 (21.2%)	27 (15.8%)	
	Oral public health	11 (6.5%)	7 (4.1%)	
	Oral pediatrics	11 (6.5%)	7 (4.1%)	
	Prosthodontics	13 (7.6%)	19 (11.1%)	
	Orthodontics	25 (14.7%)	37 (21.6%)	
	Periodontology	2 ^b^ (1.2%)	21 ^a^ (12.3%)	
	Hospitaller dentistry	5 (2.9%)	6 (3.5%)	
	Other	31 (18.2%)	22 (12.9%)	
Average workload/ week (h)	≤25 h	27 ^b^ (15.9%)	50 ^a^ (29.2%)	<0.001 *
	26–35 h	39 (22.9%)	45 (26.3%)	
	36–44 h	56 (32.9%)	55 (32.2%)	
	≥45 h	48 ^a^ (28.2%)	21 ^b^ (12.3%)	
Training sessions/ week	0	89 (52.4%)	81 (47.4%)	0.321 *
	1–2	37 (21.8%)	33 (19.3%)	
	≥3	44 (25.9%)	57 (33.3%)	
Academic training with ergonomics in clinical practice	No	104 (61.2%)	115 (67.6%)	0.213
	Yes	66 (38.8%)	55 (32.4%)	

^a,b^—different letters identify significant differences in the % of the category or in the median value of the variable within the groups (“a” higher, “b” lower), according to the * Chi^2^ test or the U of ** Mann–Whitney test, respectively. St.Dev: standard deviation.

**Table 2:** Prevalence (% and 95% confidence interval for the %) of self-reported MSDs in the last 12 months, and of avoiding the routine work in dentistry in the same period, global and by body zone, as well as the prevalence of acute MSD (last 7 days) overall and prevalence of pain complaint due to work in dentistry in the last 12 months.

	Portuguese Dentists *n* (%; 95% CI)	Italian Dentists *n* (%; 95% CI)
MSD last 12 months (any area of the body)	134 (78.8%; 95% CI: 71.9–84.7%)	140 (81.9%; 95% CI: 75.3–87.3%)
Neck	111 (65.3%; 95% CI: 57.6–72.4%)	105 (61.4%; 95% CI: 53.7–68.7%)
Shoulders	84 (49.4%; 95% CI: 41.7–56.9%)	67 (39.2%; 95% CI: 31.8–46.9%)
Thorax	31 (18.2%; 95% CI: 12.7–24.9%)	31 (18.1%; 95% CI: 12.7–24.7%)
Elbows	19 (11.2%; 95% CI: 7.3–16.8%)	27 (15.8%; 95% CI: 10.7–22.1%)
Lumbar region	89 (52.4%; 95% CI: 44.6–60.1%)	87 (50.9%; 95% CI: 43.1–58.6%)
Fists/Hands	60 (35.3%; 95% CI: 28.1–43.0%)	37 (21.6%; 95% CI: 15.7–28.6%)
Hips/thighs	21 (12.4%; 95% CI: 8.2–18.3%)	31 (18.1%; 95% CI: 12.7–24.7%)
Knees	21 (12.4%; 95% CI: 8.2–18.3%)	31 (18.1%; 95% CI: 12.7–24.7%)
Ankles and feet	20 (11.8%; 95% CI: 7.3–17.6%)	21 (12.3%; 95% CI: 7.8–18.2%)
MSD avoiding the dentistry work routine last 12 months (any area of the body)	49 (28.8%; 95% CI: 22.2–36.3%)	33 (19.3%; 95% CI: 13.7–26.0%)
Neck	27 (15.9%; 95% CI: 10.7–22.3%)	16 (9.4%; 95% CI: 5.4–14.8%)
Shoulders	21 (25%; 95% CI: 8.2–18.3%)	10 (12.7%; 95% CI: 2.8–10.5%)
Thorax	5 (2.9%; 95% CI: 1.0–6.7%)	6 (3.5%; 95% CI: 1.3–7.5%)
Elbows	5 (26.3%; 95% CI: 1.0–6.7%)	5 (17.9%; 95% CI: 1.0–6.7%)
Lumbar region	21 (12.4%; 95% CI: 8.2–18.3%)	13 (7.6%; 95% CI: 4.1–12.7%)
Fists/Hands	19 (32.2%; 95% CI: 6.9–16.9%)	7 (18.4%; 95% CI: 1.7–8.3%)
Hips/thighs	5 (2.9%; 95% CI: 1.0–6.7%)	1 (0.6%; 95% CI: 0–3.2%)
Knees	6 (3.5%; 95% CI: 1.3–7.5%)	3 (1.8%; 95% CI: 0.4–5.0%)
Ankles and feet	3 (1.8%; 95% CI: 0.4–5.1%)	3 (1.8%; 95% CI: 0.4–5.0%)
MSD last 7 days (any area of the body)	99 (58.2%; 95% CI: 50.4–65.7%)	97 (56.7%; 95% CI: 48.9–64.3%)
Presence of pain (any area of the body)	135 (79.4%; 95% CI: 72.6–85.2%)	142 (83%; 95% CI: 76.6–88.3%)

**Table 3:** Comparison between countries of the prevalence of activity-related self-reported MSD in the last 12 months and their degree of pain [0 (without pain) to 10 (maximum pain)], and prevalence of musculoskeletal disorders related to activity in dental medicine) in the last 12 months. The results indicated represent counts and % unless other statistics are indicated.

Body Area		Had Problems (Such as Pain, Discomfort or Numbness), e.g., MSD in the Last 12 Months				Degree of Pain [0 (No Pain) to 10 (Maximum Pain)]			Had to Avoid Normal Activities (in Dental Medicine) Because of Problems (MSD) in the Last 12 Months		
		Pt dentists	It dentists	*p*		Pt dentists	It dentists	*p*	Pt dentists	Pt dentists	*p*
Neck	Yes	111 (65.3%)	105 (61.4%)	0.456	Median (Q1–Q3)	6 ^a^ (5–8)	5 ^b^ (3–7)	0.001	27 (15.9%)	16 (9.4%)	0.070
	No	59 (34.7%)	66 (38.6%)		Min–Max	1–10	1–10		143 (84.1%)	155 (90.6%)	
Shoulders	Yes, right	40 ^a^ (23.5%)	14 ^b^ (8.2%)	<0.001	Median (Q1–Q3)	6 (5–8)	6 (4–7)	0.090	12 ^a^ (7.1%)	3 ^b^ (1.8%)	0.017
	Yes, left	16 ^a^ (9.4%)	9 ^a^ (5.3%)		Min–Max	1–10	1–10		4 ^a^ (2.4%)	0 ^b^ (0%)	
	Yes, both	28 ^a^ (16.5%)	44 ^b^ (25.7%)						5 ^a^ (2.9%)	7 ^a^ (4.1%)	
	No	86 ^a^ (50.6%)	104 ^a^ (60.8%)						149 ^b^ (87.6%)	161 ^a^ (94.2%)	
Thorax	Yes	31 (18.2%)	31 (18.1%)	0.980	Median (Q1–Q3)	4 (2–6)	4 (2–6)	0.893	5 (2.9%)	6 (3.5%)	0.767
	No	139 (81.8%)	140 (81.9%)		Min–Max	1–10	1–9		165 (97.1%)	165 (96.5%)	
Elbows	Yes, right	9 (5.3%)	14 (8.2%)	0.277	Median (Q1–Q3)	6 (4–8)	6 (4–7)	0.736	2 (1.2%)	3 (1.8%)	0.158
	Yes, left	7 (4.1%)	5 (2.9%)		Min–Max	1–10	2–9		3 (1.8%)	0 (0%)	
	Yes, both	3 (1.8%)	8 (4.7%)						0 (0%)	2 (1.2%)	
	No	151 (88.8%)	144 (84.2%)						165 (97.1%)	166 (97.1%)	
Lumbar region	Yes	89 (52.4%)	87 (50.9%)	0.963	Median (Q1–Q3)	6 ^a^ (5–8)	6 ^b^ (4–7)	0.034	21 (12.4%)	13 (7.6%)	0.143
	No	81 (47.6%)	84 (49.1%)		Min–Max	2–10	1–10		149 (87.6%)	158 (92.4%)	
Fists/hands	Yes, right	36 ^a^ (21.2%)	23 ^a^ (13.5%)	0.046	Median (Q1–Q3)	6 (4–8)	6 (4–7)	0.753	10 ^a^ (5.9%)	4 ^a^ (2.3%)	0.016
	Yes, left	7 ^a^ (4.1%)	5 ^a^ (2.9%)		Min–Max	1–10	3–10		7 ^a^ (4.1%)	0 ^b^ (0%)	
	Yes, both	17 ^a^ (10%)	9 ^a^ (5.3%)						2 ^a^ (1.2%)	3 ^a^ (1.8%)	
	No	110 ^a^ (64.7%)	134 ^b^ (78.4%)						151 ^b^ (88.8%)	164 ^a^ (95.9%)	
Hips/thighs	Yes	21 (12.4%)	31 (18.1%)	0.138	Median (Q1–Q3)	6 (3.75–8)	5 (2–6)	0.097	5 (2.9%)	1 (0.6%)	0.099
	No	149 (87.6%)	140 (81.9%)		Min–Max	1–10	1–10		165 (97.1%)	169 (99.4%)	
Knees	Yes	21 (12.4%)	31 (18.1%)	0.195	Median (Q1–Q3)	6 (3.5–9)	5 (4–6)	0.278	6 (3.5%)	3 (1.8%)	0.307
	No	149 (87.6%)	140 (81.9%)		Min–Max	2–9	1–9		164 (96.5%)	168 (98.2%)	
Ankle and foot	Yes	20 (11.8%)	21 (12.3%)	0.599	Median (Q1–Q3)	4 ^b^ (1–5)	5 ^a^ (4–6)	0.030	3 (1.8%)	3 (1.8%)	1.000
	No	150 (88.2%)	150 (87.7%)		Min–Max	1–8	2–10		167 (98.2%)	167 (98.2%)	

^a,b^—different letters identify significant differences in the % of the category per country (Chi^2^ test) or median value of pain level per country professionals (U of Mann–Whitney).

**Table 4:** Comparison of issues related to clinical practice in both countries.

Variables	Characteristics/Statistics	Portuguese Dentists	Italian Dentists	*p*
Breaks between appointments	Yes	68 ^b^ (40.0%)	98 ^a^ (57.3%)	0.001
Preferred working position	Standing	30 (17.6%)	23 (13.5%)	0.347
	Seated	80 (47.1%)	93 (54.4%)	
	Alternates standing/seated	60 (35.3%)	55 (32.2%)	
The most common working position regarding the patient is	Right	147 (86.5%)	133 (77.8%)	0.062
	Left	1 (0.6%)	5 (2.9%)	
	Behind	22 (12.9%)	33 (19.3%)	
Dominant work hand	Right	157 ^a^ (92.4%)	143 ^b^ (83.6%)	0.019
	Left	3 ^b^ (1.8%)	13 ^a^ (7.6%)	
	Both	10 ^a^ (5.9%)	15 ^a^ (8.8%)	
Aids of vision in clinical practice	None	127 ^a^ (74.7%)	81 ^b^ (47.4%)	<0.001
	Magnifying glasses	0 ^b^ (0%)	81 ^a^ (47.4%)	
	Microscope	43 ^a^ (25.3%)	9 ^b^ (5.3%)	
Practice of physical activity	No	89 (52.4%)	81 (47.4%)	0.357
Pain or discomfort during or after clinical activity	Yes	91 ^a^ (53.5%)	73 ^b^ (42.7%)	0.045
If Yes, after which clinical activity do you present more pain or discomfort?	Oral surgery	31 ^a^ (34.1%)	19 ^a^ (26.0%)	0.035
	Endodontics	26 ^a^ (28.6%)	20 ^a^ (27.4%)	
	Oral public health	2 ^a^ (2.2%)	0 ^a^ (0%)	
	Oral pediatrics	10 ^a^ (11%)	5 ^a^ (6.8%)	
	Prosthodontics	4 ^a^ (4.4%)	7 ^a^ (9.6%)	
	Orthodontics	8 ^a^ (8.8%)	6 ^a^ (8.2%)	
	Periodontology	2 ^b^ (2.2%)	12 ^a^ (16.4%)	
	Hospitaller dentistry	8 ^a^ (8.8%)	4 ^a^ (5.5%)	

^a,b^—different letters identify significant differences in the % of the category per country (Chi^2^ test).

**Table 5:** Risk factors (bivariate and multivariate logistics analysis) associated with the presence of self-reported MSDs in the last 12 months in dentists working in Portugal and in Italy.

	Portugal				Italy			
	Bivariate Analysis		Multivariate Analysis *		Bivariate Analysis		Multivariate Analysis **	
	*p*	OR (95% CI OR)	*p*	OR (95% CI OR)	*p*	OR (95% CI OR)	*p*	OR (95% CI OR)
Gender: Male Female	0.022	1.000	0.038	1.000	0.985	1.000		
		2.391 (1.132–5.052)		2.828 (1.057–7.568)		1.007 (0.463–2.194)		
Age group ≤30 years	0.249	1.000			0.269	1.000		
31–40 years	0.960	1.021 (0.446–2.340)			0.670	0.805 (0.296–2.186)		
41–50 years	0.059	4.426 (0.943–20.769)			0.159	3.080 (0.645–14.718)		
≥51 years	0.439	1.617 (0.478–5.468)			0.246	1.800 (0.667–4.857)		
Practice time as dentist ≤4 years	0.064	1.000	0.027	1.000	0.037	1.000	0.037	1.000
5–10 years	0.656	0.812 (0.325–2.029)	0.064	0.320 (0.096–1.069)	0.161	0.481 (0.173–1.337)	0.173	0.492 (0.177–1.366)
11–19 years	0.029	3.721 (1.147–12.076)	0.543	1.547 (0.379–6.317)	0.438	1.715 (0.439–6.704)	0.420	1.752 (0.448–6.851)
≥20 years	0.219	2.009 (0.660–6.119)	0.156	2.687 (0.687–10.518)	0.075	2.573 (0.909–7.285)	0.069	2.628 (0.927–7.446)
Average work load/week: ≤25 h	0.031	1.000	0.013	1.000	0.178	1.000		
26–35 h	0.921	0.947 (0.325–2.762)	0.593	1.442 (0.376–5.536)	0.086	2.528 (0.877–7.283)		
36–44 h	0.011	5.474 (1.477–20.290)	0.005	10.700 (2.063–55.485)	0.154	1.988 (0.773–5.110)		
≥45 h	0.664	1.263 (0.441–3.621)	0.943	0.953 (0.257–3.529)	0.106	3.694 (0.759–17.980)		
Breaks between apointments: Yes	0.013	1.000	0.026	1.000	0.759	1.000		
No		2.591 (1.222–5.495)		3.021 (1.142–7.993)		0.884 (0.404–1.935)		
Common working position: Standing	<0.001	1.000	0.003	1.000	0.285	1.000		
Seated	<0.001	7.000 (2.640–18.564)	0.002	7.064 (2.007–24.863)	0.167	2.172 (0.723–6.524)		
Alternates standing/seated	0.003	4.455 (1.690–11.744)	0.002	7.863 (2.098–29.466)	0.684	1.265 (0.409–3.913)		
Working position facing the patient: right	0.443	1.000			0.867	1.000		
Left	1.000	n.a.			0.876	0.836 (0.089–7.832)		
Behind	0.202	0.527 (0.197–1.410)			0.601	0.777 (0.301–2.004)		
Dominant Hand: right	0.280	1.000			0.447	1.000		
Left	0.110	0.138 (0.012–1.570)			0.247	0.477 (0.136–1.671)		
Both	0.999	n.a.			0.686	1.377 (0.292–6.488)		
Aids of vision: None	0.633	1.000			0.255	1.000		
Microscope		1.237 (0.516–2.969)				0.418 (0.093–1.874)		
Breaks between apointments: Yes	0.003	1.000			0.786	1.000		
Not		0.287 (0.126–0.656)				1.114 (0.510–2.434)		
Times practice physical activity: None	0.013	1.000			0.955	1.000		
1–2/week	0.027	3.593 (1.158–11.142)			0.909	0.940 (0.327–2.702)		
≥3/week	0.021	3.397 (1.207–9.561)			0.762	0.874 (0.365–2.095)		
Principal dentistry clinical activity: Oral surgery	0.650	1			0.353	1		
Endodontics	0.074	2.636 (0.911–7.633)			0.571	0.667 (0.164–2.710)		
Oral public health	0.218	2.864 (0.538–15.247)			0.999	n.a.		
Oral pediatrics	0.218	2.864 (0.538–15.247)			0.038	0.143 (0.023–0.899)		
Prosthodontics	0.978	1.018 (0.277–3.747)			0.985	1.016 (0.199–5.196)		
Orthodontics	0.123	2.545 (0.777–8.343)			0.982	0.984 (0.247–3.916)		
Periodontology	0.999	n.a.			0.249	3.810 (0.392–37.068)		
Hospitaller dentistry	0.424	2.545 (0.257–25.173)			0.346	0.381 (0.051–2.832)		
Other	0.998	n.a.			0.560	0.648 (0.150–2.794)		
Academic training with ergonomics in clinical practice: No	0.004	1			0.404	1		
Yes		4.054 (1.583–10.382)				0.709 (0.316–1.59)		
Model constant			0.003	0.090			<0.001	3.615
			AUC (95% CI) = 0.858 (0.785–0.932)				AUC (95% CI) = 0.659 (0.552–0.765)	

* Portugal: variables included in the first step in the model: gender, practice time as dentist (years), average work load/week (h), preferred working position facing the patient, and physical activity/week; ** Italy: variables included in the first step in the model: gender, practice time as dentist (years), average work load/week (h), breaks between appointments, preferred working position facing the patient, and physical activity/week. Academic training with ergonomics in clinical practice. n.a.—not applicable due to absence of data.

**Table 6:** Risk factors (bivariate and multivariate logistic analysis) associated with the presence of self-reported MSD while avoiding the routine work of dental medicine in the last 12 months, for dentists working in Portugal and in Italy.

	Portugal				Italy			
	Bivariate Analysis		Multivariate Analysis *		Bivariate Analysis		Multivariate Analysis **	
	*p*	OR (95% CI OR)	*p*	OR (95% CI OR))	*p*	OR (95% CI OR)	*p*	OR (95% CI OR)
Gender: Male Female	0.482	1.000			0.443	1.000		
		1.281 (0.642–2.557)				1.348 (0.629–2.889)		
Age group ≤30 years	0.003	1.000	0.074	1.000	0.571		0,128	1.000
31–40 years	0.893	0.940 (0.383–2.308)	0.472	0.689 (0.250–1.900)	0.649	1.296 (0.424–3.963)	0.028	38.282 (1.491–982.748)
41–50 years	0.006	3.846 (1.473–10.042)	0.039	3.701 (1.069–12.809)	0.158	2.224 (0.733–6.741)	0.060	10.271 (0.911–115.831)
≥51 years	0.016	3.526 (1.271–9.783)	0.349	1.773 (0.534–5.885)	0.610	1.286 (0.490–3.374)	0.087	3.286 (0.843–12.818)
Practice time as dentist ≤4 years	0.031	1.000	0.021	1.000	0.347		0.115	1.000
5–10 years	0.623	1.293 (0.464–3.605)	0.870	0.907 (0.283–2.907)	0.726	1.262 (0.343–4.641)	0.324	2.494 (0.406–15.320)
11–19 years	0.078	2.257 (0.914–5.576)	0.011	5.994 (1.514–23.728)	0.136	2.484 (0.750–8.224)	0.052	13.469 (0.978–185.449)
≥20 years	0.006	3.850 (1.482–10.002)	0.946	1.040 (0.334–3.241)	0.130	2.070 (0.806–5.315)	0.016	54.019 (2.110–1382.75)
Average work load/week ≤25 h	0.105	1.000			0.498	1.000		
26–35 h	0.557	1.484 (0.398–5.532)			0.190	2.095 (0.694–6.327)		
36–44 h	0.042	3.450 (1.048–11.359)			0.188	2.047 (0.705–5.944)		
≥45 h	0.090	2.875 (0.849–9.735)			0.217	2.292 (0.614–8.558)		
Breaks between apointments: Yes	0.580	1.000			0.973	1.000		
No		1.213 (0.612–2.404)				0.987 (0.458–2.127)		
Common working position: Standing	0.938	1.000			0.075	1.000	0.156	1.000
Seated	0.732	1.179 (0.460–3.017)			0.126	0.420 (0.138–1.275)	0.387	0.583 (0.172–1.979)
Alternates standing/seated	0.868	1.087 (0.406–2.911)			0.914	1.063 (0.352–3.204)	0.561	1.428 (0.430–4.746)
Working position facing the patient: right	0.532	1.000			0.124	1.000	0.130	1.000
Left	1.000	n.a.			0.999	n.a.	0.999	n.a.
Behind	0.261	0.520 (0.166–1.626)			0.041	0.212 (0.048–0.938)	0.043	0.202 (0.043–0.953)
Dominant Hand: Right	0.751	1.000			0.417	1.000		
Left	0.999	n.a.			0.999	n.a.		
Both	0.449	1.659 (0.447–6.159)			0.186	0.248 (0.031–1.957)		
Aids of vision: None	0.001	1.000			0.237	1.000		
Microscope		3.375 (1.626–7.006)				0.719 (0.082–6.278)		
Engage in any physical activity: Yes	0.825	1.000			0.194	1.000		
Not		0.928 (0.478–1.802)				1.664 (0.772–3.584)		
Times practice physical activity: None	0.142	1.000	0.020		0.233	1.000		
1–2/week	0.174	1.745 (0.782–3.895)	0.086	2.890 (0.862–9.690)	0.090	0.326 (0.090–1.189)		
≥3/week	0.344	0.658 (0.277–1.565)	0.012	4.151 (1.366–12.615)	0.560	0.780 (0.339–1.798)		
Principal dentistry clinical activity: Oral surgery	0.985	1			0.786	1		
Endodontics	0.609	1.3 (0.475–3.555)			0.892	0.909 (0.229–3.612)		
Oral public health	0.587	1.486 (0.356–6.2)			0.733	0.667 (0.065–6.871)		
Oral pediatrics	0.526	0.578 (0.106–3.153)			0.733	0.667 (0.065–6.871)		
Prosthodontics	0.838	1.156 (0.289–4.618)			0.383	1.846 (0.466–7.316)		
Orthodontics	0.742	0.821 (0.254–2.652)			0.878	1.103 (0.315–3.868)		
Periodontology	0.999	n.a.			0.755	1.25 (0.307–5.085)		
Hospitaller dentistry	0.577	1.733 (0.251–11.967)			0.853	0.8 (0.076–8.474)		
Other	0.910	1.064 (0.367–3.084)			0.145	0.19 (0.02–1.776)		
Academic training with ergonomics in clinical practice: No	0.302	1			0.584	1		
Yes		1.426 (0.727–2.797)				1.249 (0.564–2.768)		
Model constant			0.750	1.182			0.003	0.006
			AUC (95% CI) = 0.585 (0.491–0.679)				AUC (95% CI) = 0.743 (0.646–0.839)	

* Portugal: variables included in the first step in the model: age group (years), practice time as dentist (years), average work load/week(h), preferred working position facing the patient, and physical activity/week; ** Italy: variables included in the first step in the model: age group (years), practice time as dentist (years), average work load/week (h), common working position, preferred working position facing the patient, and physical activity/week. n.a.—not applicable due to absence of data.

## Data Availability

The data that support the findings of this study are available from the corresponding author upon reasonable request.

## References

[1] Pîrvu C., Pătraşcu I., Pîrvu D., Ionescu C. (2014). The dentist’s operating posture—Ergonomic aspects. J. Med. Life.

[2] Pîrvu C., Axante A., Preoteasa C., Pârlătescu J., Cărămidă M., Pîrvu D. (2021). Maintaining a Balanced Posture by Dentists—A Challenge of the Current Practice. Timis. Med..

[3] Sakzewski L., Naser-ud-Din S. (2015). Work-related musculoskeletal disorders in Australian dentists and orthodontists: Risk assessment and prevention. Work.

[4] Gandolfi M.G., Zamparini F., Spinelli A., Risi A., Prati C. (2021). Musculoskeletal Disorders among Italian Dentists and Dental Hygienists. Int. J. Environ. Res. Public Health.

[5] Kuorinka I., Jonsson B., Kilbom A., Vinterberg H., Biering-Sørensen F., Andersson G., Jørgensen K. (1987). Standardised Nordic questionnaires for the analysis of musculoskeletal symptoms. Appl. Ergon..

[6] Ramírez-Sepúlveda K.A., Gómez-Arias M.Y., Agudelo-Suárez A.A., Ramírez-Ossa D.M. (2022). Musculoskeletal disorders and related factors in the Colombian orthodontists’ practice. Int. J. Occup. Saf. Ergon..

[7] Rambabu T., Suneetha K. (2014). Prevalence of work related musculoskeletal disorders among physicians, surgeons and dentists: A comparative study. Ann. Med. Health Sci. Res..

[8] Akesson I., Johnsson B., Rylander L., Moritz U., Skerfving S. (1999). Musculoskeletal disorders among female dental personnel—Clinical examination and a 5-year follow-up study of symptoms. Int. Arch. Occup. Environ. Health.

[9] Rickert C., Fels U., Gosheger G., Kalisch T., Liem D., Klingebiel S., Schneider K.N., Schorn D. (2021). Prevalence of Musculoskeletal Diseases of the Upper Extremity Among Dental Professionals in Germany. Risk Manag. Healthc Policy.

[10] Ohlendorf D., Naser A., Haas Y., Haenel J., Fraeulin L., Holzgreve F., Erbe C., Betz W., Wanke E.M., Brueggmann D. (2020). Prevalence of Musculoskeletal Disorders among Dentists and Dental Students in Germany. Int. J. Environ. Res. Public Health.

[11] Rafie F., Zamani Jam A., Shahravan A., Raoof M., Eskandarizadeh A. (2015). Prevalence of Upper Extremity Musculoskeletal Disorders in Dentists: Symptoms and Risk Factors. J. Environ. Public Health.

[12] Garcia P.P.N.S., Gottardello A.C.A., Wajngarten D., Presoto C.D., Campos J.A.D.B. (2017). Ergonomics in dentistry: Experiences of the practice by dental students. Eur. J. Dent. Educ..

[13] Al-Ali K., Hashim R. (2012). Occupational health problems of dentists in the United Arab Emirates. Int. Dent. J..

[14] Morse T., Bruneau H., Dussetschleger J. (2010). Musculoskeletal disorders of the neck and shoulder in the dental professions. Work.

[15] Chenna D., Pentapati K.C., Kumar M., Madi M., Siddiq H. (2022). Prevalence of musculoskeletal disorders among dental healthcare providers: A systematic review and meta-analysis. F1000Research.

[16] Butera A., Maiorani C., Fantozzi G., Bergamante F., Castaldi M., Grassi R., Leuter C., Scribante A., Nardi G.M. (2024). Musculoskeletal Disorders in the Clinical Practice of Dental Hygienists and Dentists, Prevention and Awareness among Italian Professionals: Focus on Enlarging Systems. Clin. Pract..

[17] Esteves M.R.O.M. (2016). Relationship Between Happiness, Stress and Musculoskeletal Disorders in Portuguese Dentists. Ph.D. Thesis.

[18] de Almeida M.B., Moleirinho-Alves P., Oliveira R. (2024). Work-related musculoskeletal disorders among dental students: A cross-sectional study integrating the pain adaptation model. J. Public Health.

[19] Samat R.A., Shafei M.N., Yaacob N., Yusoff A. (2011). Prevalence and associated factors of back pain among dental personnel in the north eastern state of Malaysia. Int. J. Collab. Res. Intern. Med. Public Health.

[20] Macrì M., Flores N.V.G., Stefanelli R., Pegreffi F., Festa F. (2023). Interpreting the prevalence of musculoskeletal pain impacting Italian and Peruvian dentists likewise: A cross-sectional study. Front. Public Health.

[21] von Elm E., Altman D.G., Egger M., Pocock S.J., Gøtzsche P.C., Vandenbroucke J.P., STROBE Initiative (2008). The Strengthening the Reporting of Observational Studies in Epidemiology (STROBE) statement: Guidelines for reporting observational studies. J. Clin. Epidemiol..

[22] Eysenbach G. (2012). Correction: Improving the Quality of Web Surveys: The Checklist for Reporting Results of Internet E-Surveys (CHERRIES). J. Med. Internet Res..

[23] Mesquita C.C., Ribeiro J.C., Moreira P. (2010). Portuguese version of the standardized Nordic musculoskeletal questionnaire: Cross cultural and reliability. J. Public Health.

[24] Gobba F., Ghersi R., Martinelli S., Richeldi A., Clerici P., Grazioli P. (2008). Traduzione in lingua italiana e validazione del questionario standardizzato Nordic IRSST per la rilevazione di disturbi muscoloscheletrici [Italian translation and validation of the Nordic IRSST standardized questionnaire for the analysis of musculoskeletal symptoms]. Med. Lav..

[25] Hodacova L., Sustova Z., Cermakova E., Kapitan M., Smejkalova J. (2015). Self-reported risk factors related to the most frequent musculoskeletal complaints among Czech dentists. Ind. Health.

[26] Valachi B. (2008). Musculoskeletal health of the woman dentist: Distinctive interventions for a growing population. J. Calif. Dent. Assoc..

[27] Leggat P.A., Smith D.R. (2006). Musculoskeletal disorders self-reported by dentists in Queensland, Australia. Aust. Dent. J..

[28] Yi J., Hu X., Yan B., Zheng W., Li Y., Zhao Z. (2013). High and specialty-related musculoskeletal disorders afflict dental professionals even since early training years. J. Appl. Oral. Sci..

[29] Rana M.A., Ahmed S.S., Awan N., Siddique N. (2024). Are we straining to succeed? Prevalence of work-related musculoskeletal disorders among dentists in teaching hospitals. J. Pak. Med. Assoc..

[30] Gupta A., Ankola A.V., Hebbal M. (2013). Dental ergonomics to combat musculoskeletal disorders: A review. Int. J. Occup. Saf. Ergon..

[31] Garbin A.J.Í., Soares G.B., Arcieri R.M., Garbin C.A.S., Siqueira C.E. (2017). Musculoskeletal disorders and perception of working conditions: A survey of Brazilian dentists in São Paulo. Int. J. Occup. Med. Environ. Health.

[32] Singh L.P. (2018). Prevalence of Musculoskeletal Disorders Risk among Dentist: A Study in Northern India. Ergon. Int. J..

[33] Kumar D.K., Rathan N., Mohan S., Begum M., Prasad B., Prasad E.R. (2014). Exercise prescriptions to prevent musculoskeletal disorders in dentists. J. Clin. Diagn. Res..

[34] Uppada U.K., Susmitha M., Ullah Hussaini S.W., Virk I., Yadav T.G., Khader M.A. (2020). Ergonomics among dentists in the states of Telangana and Andhra Pradesh. Natl. J. Maxillofac. Surg..

[35] Mclean L., Tingley M., Scott R.N., Rickards J. (2001). Computer terminal work and the benefit of microbreaks. Appl. Ergon..

[36] Pispero A., Marcon M., Ghezzi C., Massironi D., Varoni E.M., Tubaro S., Lodi G. (2021). Posture Assessment in Dentistry for Different Visual Aids Using 2D Markers. Sensors.

[37] Zarra T., Lambrianidis T. (2014). Musculoskeletal disorders amongst Greek endodontists: A national questionnaire survey. Int. Endod. J..

[38] Aghahi R.H., Darabi R., Hashemipour M.A. (2018). Neck, back, and shoulder pains and ergonomic factors among dental students. J. Educ. Health Promot..

[39] Feige S., Holzgreve F., Fraeulin L., Maurer-Grubinger C., Betz W., Erbe C., Nienhaus A., Groneberg D.A., Ohlendorf D. (2024). Ergonomic Analysis of Dental Work in Different Oral Quadrants: A Motion Capture Preliminary Study among Endodontists. Bioengineering.

[40] Benfaida S., Hachami I., Chafik R., Hamza M., Bennani A. (2024). Musculoskeletal Disorders among Dentists in the Private Sector. Eur. J. Med. Health Sci..

[41] Sullivan G.M., Feinn R. (2012). Using Effect Size-or Why the P Value Is Not Enough. J. Grad. Med. Educ..

[42] Buoite S.A., Cargnel A., Raffini A., Mazzari L., Martini M., Ajčević M., Accardo A., Deodato M., Murena L. (2024). Shoulder Tensiomyography and Isometric Strength in Swimmers Before and After a Fatiguing Protocol. J. Athl. Train..

[43] Parmar A., Scott M., Brand C., Jones T.W. (2020). An assessment of the contractile properties of the shoulder musculature in elite volleyball players using tensiomyography. Int. J. Sports Phys. Ther..

